# Current Status of Porcine Reproductive and Respiratory Syndrome Vaccines

**DOI:** 10.3390/vaccines12121387

**Published:** 2024-12-10

**Authors:** Honglei Wang, Wenhai Feng

**Affiliations:** 1 Department of Clinical Laboratory, Affiliated Hospital of Hebei University, Baoding 071000, China; 2 State Key Laboratory of Agrobiotechnology, China Agricultural University, Beijing 100193, China; 3 Ministry of Agriculture Key Laboratory of Soil Microbiology, China Agricultural University, Beijing 100193, China; 4 Department of Microbiology and Immunology, College of Biological Sciences, China Agricultural University, Beijing 100193, China

**Keywords:** porcine reproductive and respiratory syndrome virus (PRRSV), vaccine, PRRS modified live virus (MLV)

## Abstract

Porcine reproductive and respiratory syndrome (PRRS), characterized by reproductive failures in breeding pigs and respiratory diseases in growing pigs, is a widespread and challenging disease. The agent, PRRSV, is a single-strand RNA virus that is undergoing continuous mutation and evolution, resulting in the global spread of multiple strains with different genetic characteristics and variable antigens. There are currently no effective measures to eradicate PRRS, and vaccination is crucial for controlling the disease. At present, various types of vaccine are available or being studied, including inactivated vaccines, modified live virus (MLV) vaccines, vector vaccines, subunit vaccines, DNA vaccines, RNA vaccines, etc. MLV vaccines have been widely used to control PRRSV infection for more than 30 years since they were first introduced in North America in 1994, and have shown a certain efficacy. However, there are safety and efficacy issues such as virulence reversion, recombination with field strains, and a lack of protection against heterologous strains, while other types of vaccine have their own advantages and disadvantages, making the eradication of PRRS a challenge. This article reviews the latest progress of these vaccines in the prevention and control of PRRS and provides scientific inspiration for developing new strategies for the next generation of PRRS vaccines.

## 1. Introduction

Porcine reproductive and respiratory syndrome (PRRS) is a severe infectious disease caused by the PRRS virus (PRRSV), which is widespread across all countries with pigs. The disease is characterized by massive reproductive failures in pregnant sows and respiratory disorders in growing pigs, imposing a huge economic burden on the global pig industry over the past 30 years. It is estimated that the total cost to U.S. swine producers is approximately USD 664 million annually. The economic losses caused by PRRSV are EUR 3–160 per sow in Europe and CNY 1424.37 (CNY 1049.78–1609.70) per sow from breeding to fattening in China [1,2]. The data stress the necessity to control PRRS effectively.

PRRSV is classified into two genotypes: the European type (PRRSV-1) and the American type (PRRSV-2), isolated from the Netherlands in 1991 (Lelystad virus (LV) isolation) and the United States in 1992 (VR-2332 isolation), respectively. According to the classification system, PRRSV-1 is divided into three subtypes (subtypes 1–3), and PRRSV-2 is divided into 9 lineages with several sublineages of each lineage. Despite having similar disease phenotypes, clinical symptoms, and genomic structures, the nucleotide homology between the two types is only about 60%. The virus includes at least 16 non-structural proteins (nsps, nsp1α, nsp1β, nsp2N, nsp2TF, nsp2-6, nsp7α, nsp7β, and nsp8-12) and 8 structure proteins (glycoprotein (GP) 2–5, ORF5a, envelope (E), membrane (M), and nucleocapsid (N) protein) [3,4]. GP5 is the primary inducer of neutralizing antibodies, and GP2, GP3, and GP4 also possess virus-neutralizing B cell epitopes, while GP3, GP4, GP5, M, N, and some non-structural proteins possess potential T cell epitopes. Exposing the viral immunogenic proteins in complexes to efficiently induce specific and protective B- and T cell responses in innovative vaccine formulations is critical to eliciting effective anti-PRRSV immunity.

As a single plus-stranded RNA virus, PRRSV evolution is occurring continuously in the swine population, resulting in the emergence of novel strains with different virulence and antigenicity. For example, an “atypical” or “acute” variant appeared in Iowa, USA, in the middle of the 1990s, and a new PRRSV strain called MN184 was isolated in Minnesota, USA, in 2001, although the vaccine was used in the late 1990s [5]. In China, the emergence of a highly pathogenic PRRSV (HP-PRRSV) strain with a 30-amino-acid (aa) deletion in nsp2 led to atypical PRRS pandemics in 2006, which caused high fever, high morbidity, high mortality, and devastated the Chinese swine industry [6]. In 2008, a NADC30 strain associated with severe respiratory disease was reported in Iowa. Interestingly, since 2013, several PRRSV-2 field isolates have been identified in China with high nucleotide similarity to NADC30 strain, ranging from 93.2% to 95.8%, and have been designated as NADC30-like PRRSVs. In 2014, additional strains that resulted from the recombination of HP-PRRSV and NADC-30-like strains caused several outbreaks in China [7]. Another strain, named NADC34, and characterized by a continuous deletion of 100 aa in the nsp2-coding region, has been present in America since 2004. Then, a similar strain, NADC34-like PRRSV, was discovered in China and has undergone complex recombination with local strains since 2017 [8]. Large mutations have also occurred in the European type. In the 1990s, genetically mutated PRRSV-1 strains, subtypes 2 (Bor strain) and 3 (Lena strain), with greater virulence and pathogenicity, were discovered in Russia/Belarus [9]. Since the beginning, instances of increased virulence have emerged episodically around the world. Ruedas-Torres et al. summarize the virulent PRRSV-1 and virulent PRRSV-2 strains associated with field outbreaks [10]. This poses a challenge to developing a vaccine with broad protection against the evolving PRRSV.

PRRSV-infected pigs generally exhibit inadequate immunity against PRRSV, which is characterized by a weak host innate immune response along with delayed protective antibody and cell-mediated immune (CMI) responses [11,12,13]. PRRSV may persist over several months in pigs or be “lifelong”, but the exact mechanisms of pathogenesis and immune response of PRRSV are not fully understood.

Currently, vaccination is the main means for preventing PRRS, and at least 20 vaccines, including modified live vaccines (MLVs) and inactivated virus vaccines, are commercially available globally. The existing vaccines, which mainly protect against homologous rather than heterologous infections, effectively reduce clinical morbidity and viremia [14]. Although the vaccines have been used for nearly 30 years to provide reasonable protection against PRRSV, a “perfect” PRRS vaccine is not yet available. The genetic diversity and the impaired immune response induced by PRRSV make it difficult to develop a “perfect” vaccine with broad-spectrum and highly effective protection. The key challenges for PRRS vaccines are shown in Table 1. Development of new technologies and advancements in scientific knowledge of the pathogenesis and immune mechanisms against PRRSV have sped up the development of safer and more effective vaccines, such as MLV vaccines, inactivated vaccines, subunit vaccines, live-carrier vaccines, DNA vaccines, RNA vaccines, etc. This manuscript reviews the current research status of various PRRS vaccines to provide some ideas for the development of new vaccines that can offer broad and robust protection against PRRSV infection.

## 2. Methods

This review is conducted through manual searches of several academic search engines and databases, including PubMed, Web of Science, and Google Scholar up to July 2024, and includes only published studies. We selected studies in English and excluded some studies from the search results, including case reports, conference proceedings, protocol papers, and book reviews. In addition, we screened and evaluated the titles and abstracts of the publications. Referring to the selected titles and abstracts, the full-text articles were critically appraised to determine which articles could be in this scoping review. In writing the literature review, we have referred to the chapters in review papers, and original research publications that particularly addressed the sections of this review. First, irrelevant studies were filtered out of the titles and abstracts. After that, full-text articles were examined to make sure that they satisfied our requirements for inclusion. Finally, in order to find more pertinent research that might not have been found in our first database search, we also looked through the reference lists of a few chosen papers.

## 3. PRRS MLV Vaccines

### 3.1. The Status of MLV Vaccines

Several MLV vaccines against PRRSV-1 and PRRSV-2 have been licensed and marketed in different countries depending on the genotype of the virus. The efficacy of MLVs has been demonstrated in many publications and is considered to have a protective effect against both reproductive failures and respiratory diseases, including but not limited to reducing clinical symptoms and viremia, alleviating macro- and micro-pulmonary lesions, shortening virus shedding period, and reducing secondary bacterial infections. However, the MLV vaccine proves to be more effective against homologous strains, whereas it decreases significantly against genetically unrelated heterologous PRRSV strains.

Based on the local prevalence of PRRSV strains, manufacturers isolate the strain with severe clinical symptoms or economic losses to create vaccines. The MLVs against PRRSV-1, including Ingelvac PRRSFLEX EU, Porcilis PRRS, Amervac-PRRS, Unistrain PRRS, Pyrsvac-183, ReproCyc PRRS EU, and Suvaxyn PRRS MLV, are licensed primarily in European countries and other countries where PRRSV-1 is endemic (Table 2). The Ingelvac PRRSFLEX EU vaccine is attenuated from a field virus isolated in 2002 from a farm in Germany. The vaccine has been shown to provide a good protective effect on piglets, and the inoculation of piglets as early as 2 weeks of age can reduce the appearance of clinical signs and the level of viremia [23,24,25]. In addition, it is protective against a heterologous type 1 PRRSV strain challenge [26]. ReproCyc PRRS EU is a new MLV vaccine against PRRSV-1, which was introduced to the European market in 2015 for immunizing breeding females. Multiple clinical trials have demonstrated the safety and protective efficacy of this vaccine, which can effectively reduce PRRSV viremia in piglets and gilts after homologous excitation [24,25,26,27,28]. Porcilis PRRS is another MLV vaccine for use in breeding and finishing pigs from 2 weeks of age, which has been reported to induce efficient CMI responses and protect against the attack of heterologous European field strains, with remarkable reductions in clinical symptoms in terms of incidence, duration, and severity [29,30]. In addition, Porcilis PRRS can provide partial clinical and virological protection in the face of attack by the highly pathogenic PRRSV-1 subtype 3 strain, Lena [31,32]. Unistrain PRRS is able to reduce the level of type 1 but not type 2 PRRSV-caused viremia [33]. Unistrain PRRS MLV, administered by intramuscular (IM) or intradermal (ID) injections, provides partial protection against HP-PRRSV infection as evidenced by reductions in the level of viremia and lung lesions [34]. Similarly, Suvaxyn PRRS does not provide adequate heterologous protection, but the recombinant MLV virus expressing membrane-bound IL-15 significantly boosts the response of NK and γδ T cells, as well as provides better protection against challenge with heterologous strain, NADC20 [35].

Ingvar PRRS MLV, Prevacent PRRS, Ingvar PRRSATP, Forster PRRS, etc., are developed for PRRSV-2, mainly licensed in the United States, China, and other Asian countries (Table 2). Ingelvac PRRS MLV, derived from VR2332 after continuous passages in a monkey kidney cell line, CL2621, is the first vaccine introduced to the U.S. market. Studies have shown that it effectively reduces reproductive failure in sows and alleviates clinical symptoms in piglets [36,37]. Nonetheless, a severe form of PRRS, known as acute or atypical PRRS, has been reported on farms using the MLV vaccination, highlighting the limited roles of the vaccine. However, a study shows that the MLV vaccine remained effective against recently isolated heterologous PRRSV-2 strains after 20 years of use in Korea [38]. The incidence of acute or atypical PRRS in vaccinated pigs led to the introduction of the Ingelvac PRRS ATP vaccine, which is made from an attenuated derivative of JA142 and is commercially available [36]. The MLV vaccine has been reported to provide partial protection from heterologous challenges, including HP-PRRSV, NADC30-like, and NADC34-like strains [39,40,41,42,43,44]. Vaccination with Inglevac PRRS MLV can decrease the duration of viremia and viral load, shorten the duration of hyperthermia, and reduce macroscopic lung lesions, but it cannot completely reduce the clinical signs [43]. Prevacent PRRS MLV, derived from a PRRSV isolation of lineage 1, is partially protective against heterologous challenge of lineage 1, 5, and 8 [45]. Fostera PRRS MLV is a type 2 PRRSV vaccine derived from the US isolate P129, which belongs to lineage 8. It was launched in 2012 and entered Asian markets from 2013-2014. Under experimental conditions, the vaccine can confer partial cross-protection against heterologous and phylogenetically distant strains of PRRSV-1 and PRRSV-2 (lineage-1, 3, 8, 9) infection [30,33,46,47,48,49,50]. Fostera PRRS is prepared by the passage in a porcine-originated cell line, and the specific attenuating process is hypothesized to contribute to its effectiveness in reducing viremia following infection with heterologous PRRSV strains. However, faced with a heterologous variant strain evolved from recombination between NADC30-like, NADC34-like, and JXA1-like viruses, Fostera PRRS provides only a weak cross-protection. After vaccination, there were still gross lung lesions and microscopic pathological lesions characterized by interstitial pneumonia, as well as high levels of viral load in the piglets.

Currently, commercial PRRS MLVs are widely used in Chinese swine farms for dealing with the complex PRRSV situation. TJM-F92 and JXA1-R MLVs provide efficacious protection against the HP-PRRSV TP strain infection, while VR2332 MLVs provide limited cross-protection [51]. Vaccination with the classical PRRSV (VR2332 and CH-1R) vaccines appears to provide more effective protection against NADC30-like viruses than the highly virulent vaccine [52,53]. Additionally, the protection efficacy of PRRS MLVs against heterologous, highly pathogenic NADC34-like strains was also evaluated and reported to be partially effective. Piglets inoculated with the VR2332 MLVs exhibit transient clinical symptoms, mild fever, low levels of viremia and tissue lesions. In contrast, R98 MLV-immunized piglets show more severe clinical signs after the challenge [44]. The parental strain and its lineage of the major commercial MLV vaccines have been listed in Table 2. According to the virus prevalent in local pig farms, a vaccine can be selected.

**Table 2:** Major PRRS modified-live virus (MLV) vaccines.

Species	Vaccine	Parental Strain	Lineage	Usage	Producer	Heterologous Protection
PRRSV-1	Ingelvac PRRSFLEX EU	94881	subtype 1	Piglet	Boehringer Ingelheim	Provide protection against virulent, heterologous PRRSV-1 challenge [25].
	ReproCyc PRRS EU	94881	subtype 1	Sow	Boehringer Ingelheim	Reduce viremia, fetal damage, and transplacental transmission caused by a heterologous PRRSV-1 strain [28].
	Unistrain PRRS	VP-046 BIS	subtype 1	Piglet, Sow	Hipra	Provide partial protection when the pigs are challenged the subtype 3 PRRSV strain, Lena [31]; can reduce the levels of type 1 but not type 2 PRRSV viremia [33]; only provided partial protection against HP-PRRSV [34].
	Amervac PRRS	VP-046	subtype 1	Piglet, Sow	Hipra	Induce relatively slow cell-mediated immunity (CMI), increase production of IL-10, and low lung lesions against co-challenge with PRRSV-1 and PRRSV-2 [54].
	Porcilis PRRS	DV	subtype 1	Piglet, Sow	MSD Animal Health	Induce efficient CMI responses and had a protective effect on a heterologous European (Italian cluster) field strain [29,30]; pProvide partial protection when the pigs are challenged the subtype 3 PRRSV strain, Lena [31]; iInduce relatively slow CMI, increased production of IL-10, and low lung lesions against co-challenge with PRRSV-1 and PRRSV-2 [54].
	Suvaxyn PRRS MLV	96V198	subtype 1	Piglet, Sow	Zoetis	Unable to mount sufficient heterologous protection against heterologous challenge with the PRRSV NADC20 strain [35].
PRRSV-2	Prevacent PRRS	RFLP 184	lineage 1	Piglet, Sow	Elanco	Can partially protect against heterologous and phylogenetically distant strains of PRRSV-2, including NC174 or NADC30 (both lineage 1), VR2332 (lineage 5), or NADC20 (lineage 8) [45].
	Ingelvac PRRS MLV	VR-2332	lineage 5	Piglet, Sow	Boehringer Ingelheim	Provide a limited cross-protection against the HP-PRRSV strain or NADC34-like strain infection [44,51,55]; induce relatively slow CMI, increased production of IL-10, and low lung lesions against co-challenge with PRRSV-1 and PRRSV-2 [54].
	R98	R98	lineage 5	Piglet, Sow	Nanjing Agricultural University	Is not effective against NADC34-like strains [44].
	Prime Pac PRRS	Neb-1	lineage 7	Piglet, Sow	MSD Animal Health	Induce relatively slow CMI, increased production of IL-10, and low lung lesions against co-challenge with PRRSV-1 and PRRSV-2 [54].
	Ingelvac PRRS ATP	JA-142	lineage 8	Piglet, Sow	Boehringer Ingelheim	Induce relatively slow CMI, increased production of IL-10, and low lung lesions against co-challenge with PRRSV-1 and PRRSV-2 [54].
	Fostera PRRS	P129	lineage 8	Piglet, Sow	Zoetis	Confer partial cross-protection against heterologous challenge of PRRSV-1 and PRRSV-2 (lineage-1 (SNUVR090851), 3, 8 (HP-PRRSV), 9 (NADC20)) strains under experimental conditions [30,33,46,47,48,49,50]; provide incomplete cross-protection against a heterologous variant strain that resulted from the recombination between NADC30-like, NADC34-like, and JXA1-like viruses [56]; induce relatively slow CMI, increase production of IL-10, and low lung lesions against co-challenge with PRRSV-1 and PRRSV-2 [54].
	CH-1R	CH-1a	lineage 8	Piglet, Sow	Harbin Veterinary Research Institute,	Provide better cross-protection than HP-PRRSV MLV vaccines against NADC30-like strains [52,53].
	HuN4-F112	HuN4	lineage 8	Piglet, Sow	Harbin Veterinary Research Institute,	Cannot provide full protection against a recombinant virus from lineage 8 and 3 [55].
	JXA1-R	JXA1	lineage 8	Piglet, Sow	Chinese Center for Animal Disease Control and Prevention	Provide efficacious protection against the HP-PRRSV strain infection [51]; can confer protection against the heterologous PRRSV strain NADC-20 [57]; provide limited cross-protection against NADC30-like strain [53].
	TJM-F92	TJ	lineage 8	Piglet, Sow	Institute of Special Animal and Plant Sciences, CAAS	Provide efficacious protection against the HP-PRRSV strain infection [51]; provide limited cross-protection against NADC30-like strain [53,58].

### 3.2. The Challenges of MLV Vaccines

Although PRRSV MLV vaccines have been licensed in multiple countries based on the prevailing genotypes for nearly 30 years, some concerns cannot be ignored, including the fact that these vaccines do not provide complete protection against the emerging PRRSV subtype and are not safe enough during use (Table 1). The challenge of safety arises for PRRSV MLVs due to vertical transmission and persistent infections in vaccinated hosts. The vaccine virus has been observed to cross the placental barrier in pregnant sows, thereby infecting the developing fetuses and subsequently transmitting the virus to naive newborn piglets during lactation [17,18]. A study has shown that administering the MLVs to pregnant sows on the 100th day of gestation resulted in congenital infection and viremia in 10% of piglets, highlighting the potential risks of vaccination during late gestation [17]. In addition, boar vaccination using commercial MLV vaccines cannot decrease the subsequent shedding of PRRSVs, which may lead to the spread of vaccine viruses to unvaccinated animals [15,16]. Multiple PRRS outbreaks have occurred in vaccinated or unvaccinated swine herds. Based on genomic sequencing and restriction fragment length polymorphism patterns, the only infectious agent detected in some field cases of severe reproductive failures in breeding herds and respiratory diseases in growing pigs is a PRRSV isolate that is highly homologous to the MLV vaccines used in the herd. Finally, vaccine viruses may also recombine with field strains or other vaccine strains, resulting in the creation of potentially new genetically distinct variants of PRRSV, which may contribute to virulence and disease incidence. Indeed, there are several reports about the attenuated vaccine reverting to a virulent type [59]. These raise significant concerns about vaccine efficacy and safety.

The reorganization is no accident. In European countries, recombination between the PRRS MLV viruses and PRRSV-1 field strains has been documented (Table 3). For example, a vaccine-derived PRRSV strain genetically closely related to Unistrain PRRS MLV was isolated in Hungary in 2016 and three recombinant PRRSV strains (GER18-258 from Germany, AUT22-97 and AUT20-1664 from Austrian) that cause mild to severe reproductive and respiratory diseases are all derived from the Ingelvac PRRSFLEX EU vaccine [59]. Additionally, the Chinese pig herd has also shown evidence of recombination between the vaccination strain and PRRSV-1 wild-type strain. An example is the isolation of HLJB1, which is recombined from the Amervac vaccine and the BJEU06-1 isolate [60].

In the United States and many Asian countries, including China and South Korea, novel pathogenic PRRSV-2 strains recombined from wild-type and PRRS MLV strains have been reported (Table 3). For instance, the isolation, Em2007, is a natural recombinant of the vaccine strain CH-1R and the circulating HP-PRRSV strain WUH1. The virus, with a unique deletion of 68 amino acids in nsp2, induces distinct clinical signs, including blue ears, inappetence, lethargy, persistent high fever, red discolorations in the body, mild interstitial pneumonia, and nonsuppurative encephalitis [61]. GM2, a new variant derived from the recombinant of Ingelvac PRRS MLV and QYYZ-like strain, is significantly more virulent than either QYYZ strain or Ingelvac PRRS MLV. This is because the GM2 strain has a much higher reproduction capacity than its parent strains in vivo [62,63]. RespPRRS MLV recombines with NADC30-like and JXA1-like strains to generate a new variant, SCN17, which causes a persistent fever, moderate interstitial pneumonia, and increased viremia in piglets [64]. FJWQ16 and FJXS15 strains, which are derived from HP-PRRS MLVs and NADC30 virus, show 20% and 25% mortality in pigs, respectively [41,65]. RespPRRS MLV, JXA1-P80, JXA1-R, and Fostera PRRS MLV are reported to recombine with the NADC30-like strain in China and Korea. The recombinant strain PRRSV-HB-16-China-2019, TJnh1501, FJXS15, and 21R2-63-1 inoculated piglets exhibit persistent fever, obvious respiratory symptoms, and significant pathological lung damage [65,66,67,68,69,70].

Instances of recombination between two PRRSV MLV strains have also been reported in the USA, Denmark, France, and China [71]. PRRS-FR-2014-56-11-1, isolated in France, has been identified as a recombinant between the Unistrain and Porcilis vaccines [72]. The USA/IN105404/2021 isolate obtained from a pig in Indiana is a natural recombinant isolate from Ingelvac PRRS MLV and Prevacent PRRS, and a recombinant strain from Amervac-PRRS MLV and Porcilis PRRS MLV was isolated in pig herds in China [60]. In addition, the PRRSV ‘Horsens’ strain has emerged as a recombinant between Amervac-PRRS MLV (ORFs 3–7) and Suvaxyn PRRS MLV (ORFs 1–2 and part of ORF 3), causing severe disease in infected herds in Denmark [73]. The recombinant strain has regained substantial virulence even though both of its parental strains are attenuated vaccine viruses [73]. These reports provide evidence for recombination between the MLV vaccine virus and a circulating virus, and the recombination events potentially lead to virulence reversion. However, the current vaccine may not be effective against the recombinant strain. For example, neither VR2332 MLV nor HuN4-F112 provides full protection against ZJnb16-2, a recombinant PRRSV-2 Strain from lineages 8 and 3 in China [55].

Therefore, improving safety and efficacy should be a major consideration in the design and development of the PRRS vaccine. Immunization strategies should be reformulated. For example, the MLV vaccines are used on farms that have experienced PRRS outbreaks. Contemporary advanced biotechnologies including PRRSV cDNA clones, de-optimization of codon pairs, chimeric PRRSVs, and DNA shuffling may be helpful. The methods are mentioned in Table 1.

**Table 3:** A partial report of natural recombination events of PRRSV vaccines.

Vaccine Strain	Wild or Vaccine Strain	Recombinant Virus	Recombinant Virulence	Place	References
Ingelvac PRRSFLEX EU MLV	PRRSV-1 subtype 1	GER18-258	Leads to symptoms such as, conjunctivitis, enlarged joints, respiratory distress, stillbirths, weak piglets born, and elevated pre-weaning mortality	Germany	[59]
Ingelvac PRRSFLEX EU MLV	PRRSV-1 subtype 1	AUT20-1664 and AUT22-97	Respiratory distress, wasting, and increased mortality	Austrian	[59]
Amervac-PRRS MLV	PRRSV-1 subtype 1	HLJB1	Cause fever, reddened conjunctiva, and respiratory symptoms	China	[60]
CH-1R	PRRSV-2 lineage 8 (HP-PRRSV WUH1 strain)	Em2007	Obvious clinical signs, including inappetence, lethargy, high and continuous fever, red discolorations in the body, and blue ears; mild interstitial pneumonia and nonsuppurative encephalitis; no deaths	China	[61]
RespPRRS MLV/Ingelvac PRRS MLV	PRRSV-2 lineage 3 (QYYZ)	GM2	Induce severe respiratory problems, diarrhea, poor growth and persistent higher fever	China	[62,63]
RespPRRS MLV	PRRSV-2 lineage 1 (NADC30-like) and 8 (HP-PRRSV JXA1-like strain)	SCN17	Exhibit moderate virulence, causing a persistent fever, moderate interstitial pneumonia, high level of viremia and antibodies	China	[64]
HP-PRRSV MLV (JXA1-R)	PRRSV-2 lineage 1 (NADC30-like)	FJXS15	Cause severe histopathological lung lesions and is highly virulent	China	[65]
RespPRRS MLV	PRRSV-2 lineage 1 (NADC30-like)	PRRSV-HB-16-China-2019	Is moderately virulent	China	[66]
HP-PRRSV MLV (JXA1 P80)	PRRSV-2 lineage 1 (NADC30-like)	TJnh1501, SDyt1401, SDwh1601	Cause a protracted fever, moderate respiratory symptoms, elevated viremia, and visible lung lesions in piglets	China	[67,68]
HP-PRRSV MLV (JXA1-R)	PRRSV-2 lineage 1 (NADC30) and 8 (HP-PRRSV TJ strain)	DJY-19	Cause high fever and other symptoms of PRRS	China	[70]
Ingelvac PRRS MLV	Prevacent PRRS	USA/IN105404/2021	Induce microscopic lesions	Indiana, USA	[71]
Unistrain PRRS MLV	Porcilis PRRS MLV	PRRS-FR-2014-56-11-1, PRRS-FR-2016-56-11-1	No significant clinical signs; increased excretion and transmission capacities compared to parental vaccine strains	France	[72]
Amervac-PRRS MLV	Suvaxyn PRRS MLV (96V198 strain)	“Horsens” virus	Highly transmissible; cause severe disease in infected herds	Danish	[73]
Ingelvac PRRS MLV	PRRSV-2 lineage 8 (HP-PRRSV JXA1 similar strain)	SD2020	Cause high fever, dyspnea, and depression, with a mortality rate of 60%	Shandong, China	[74]
Prevacent MLV	PRRSV-2 lineage 1	5606R-S6-L001/IA/2015/ISU-9	Cause typical symptoms	USA	[75]
Fostera PRRSV vaccine	PRRSV-2 Lineage 1 (IA76950-WT)	IA70388-R	Exhibit coughing and had interstitial pneumonia	Iowa, USA	[76]
Fostera PRRS MLV	PRRSV-2 lineage 1	20D160-1, 21R2-63-1	Causes pigs to develop abdominal breathing and coughing	Korea	[77]
HP-PRRSV MLV (TJM-F92)	PRRSV-2 lineages 1 (NADC30-like), 3 (QYYZ-like), and 8.7 (HP-PRRSV JXA1-like strain)	PRRSV2/CN/SS0/2020, PRRSV2/CN/SS1/2021, PRRSV2/CN/L3/2021, PRRSV2/CN/L4/2020	Is high pathogenicity in pigs	China	[78]
HP-PRRSV MLV (TJM-F92)	HP-PRRSV MLV (HuN4-F112)	HeN1301	Mild clinical symptoms	China	[79]
Amervac-PRRS MLV	Porcilis PRRS MLV (DV strain)	TZJ2134	Cause mild clinical symptoms	China	[80]

## 4. Inactivated Vaccines

Inactivated PRRSV vaccines have been licensed globally due to that they are safer and more stable than MLV vaccines. However, because of their inadequate protection, the inactivated vaccines have not been accessible in the US since 2005. The inactivation causes the denaturation of viral proteins or damage to their genome, which impairs cellular and mucosal protection and produces temporary immunity.

Indeed, there are reports showing that the inactivated vaccine cannot induce the detectable production of PRRSV-specific antibodies and CMI responses against wild-type virus infection, which are not effective in clearing PRRSV [81,82]. Therefore, the most important thing is to improve the inactivated PRRSV vaccine-induced immune efficacy.

Many studies have investigated the measures to enhance the efficacy of inactivated PRRSV vaccines (Table 4). The development of inactivated PRRSV vaccines uses inactivation techniques that preserve viral entry-associated domains, such as treatment with ultraviolet (UV) radiation and treatment with binary ethylenimine (BEI), which induce virus-specific antibodies and strongly prime the virus-neutralizing (VN) antibody responses [83]. Vaccine adjuvants can facilitate antigen uptake and presentation by antigen-presenting cells (APCs), as well as activate innate immune receptors for cytokine production and maturation/migration of dendritic cells (DCs) to improve protective capabilities. This, in turn, reduces the amount of immune substances needed, lowering the cost of vaccine manufacturing. The adjuvants, including emulsion, polysaccharide, IFNα, IFNγ, GM-CSF, and toll-like receptor agonists, are reported to enhance the immune responses of inactivated PRRSV vaccines. Radix pseudostellariae polysaccharide (RPP) increases the immune response of the inactivated PRRSV vaccine through interactions with the microbiome and metabolome [84,85,86,87,88,89]. A PRRSV-specific IgM monoclonal antibody (Mab)-PR5nf1 as vaccine adjuvants can enhance PRRSV inactivated vaccine-mediated CMI and survival rate, which are even higher than those in the MLV group [90].

Nanoparticle (NPs)-based technologies have attracted great interest in the development of novel vaccine candidates over the past decades due to their multiple advantages over inactivated viral or subunit-soluble antigens [91]. NPs have adjuvant-like functions that can enhance antigen adsorption and uptake by APCs, promote antigen processing, induce maturation of DCs, and facilitate the cross-presentation of antigens to CD8 T cells via major histocompatibility complex (MHC) class I. Additionally, NPs can induce the production of various innate cytokines, thereby modulating the humoral and cellular immune responses [92]. Thus, the inclusion of an inactivated virus in NPs may improve the efficacy of inactivated PRRSV vaccines. Inactivated PRRSV loaded into polylactic acid (PLA) nanoparticles can improve immune responses and protective efficacy. Chaikhumwang et al. reported that PLA nanoparticles encapsulating inactivated PRRSV combined with heat-unstable enterotoxin subunit B (LTB) and dimethyl dioctadecyl ammonium bromide (DDA) are effective at eliciting immune responses against PRRSV and offering protection from infection [93]. Additionally, with entrapped inactivated PRRSV, mannose-modified gelatin nanoparticles, MnGNP, can effectively and precisely target monocyte-derived dendritic cells (MoDCs) for maturation and activation, which subsequently enhances T cell activation, proliferation, and function to eliminate PRRSV-infected cells [94]. Entrapping the killed PRRSV antigens (KAg) in biodegradable PLGA [poly(d,l-lactide-co-glycolide)] nanoparticles (NP-KAg) can elicit an immune response against PRRSV after being administered intranasally to pigs [95,96]. In pigs vaccinated with NP-KAg and subjected to homologous virus attack, viremia was completely cleared within 2 weeks, and the virus neutralization titer significantly increased in the lungs, which shows that NP-KAg can induce cross-protective immune response [97]. In order to further strengthen immunogenicity, the NP-KAg is coupled with the whole-cell lysate of Mycobacterium tuberculosis (M. tb WCL), a potent mucosal adjuvant. The vaccine formulation significantly clears the viremia caused by heterologous PRRSV. Immunologically, strong humoral immune responses, characterized by high-affinity antibodies that enhance viral neutralization titers, and cell-mediated immune responses, indicated by an increased number of IFN-γ-secreting CD4^+^ and CD8^+^ lymphocytes and decreased secretion of immunosuppressive cytokines, are observed in the lungs [98].

There are two to five possible N-linked glycosylation sites in the ectodomain of PRRSV GP5 protein, and it has been proposed that glycans surrounding the primary neutralizing epitopes reduce the immunogenicity of PRRSV. De-glycosylation of GP5 has been reported to increases the immunogenicity of the inactivated PRRSV, and two doses of the modified inactivated virus provide protection against the homologous challenge, including significantly increased neutralizing antibody titers and rapid clearing of viremia [99]. Furthermore, using an inactivated vaccine and performing segregated rearing of the offspring are successful in eliminating PRRSV in a large-scale pig farm during the National PRRS Eradication Programme of Hungary [22]. These results indicate that inactivated PRRSV vaccines have a potential role.

**Table 4:** Some explorations of improving PRRSV inactivated vaccine-induced immune efficacy.

Measure	Specific Method	Virus Strain	Immune Efficacy	References
Optimization of inactivation procedures	UV-killed	Lelystad	Induces virus-specific antibodies and strongly prime the virus-neutralizing (VN) antibody response.	[83]
	binary ethylenimine (BEI)-killed	Lelystad	Induces high titers (3.4 log2) of VN antibodies and, after challenge, neutralizing antibody titers rise to a mean value of 5.5 log2, and the duration of the viremia is reduced to an average of 1 week.	[83]
Addition of Adjuvants	Radix pseudostellariae polysaccharide (RPP)	CH-1a	Significantly increases the concentrations of PRRSV GP5 protein antibody, interleukin (IL)-2, IL-4, IL-10, and interferon (IFN)-γ through interactions with the microbiome and metabolom.	[84]
	Emulsion	07V063	Montanide ISA28RVG induces specific antibody responses; squalene in water emulsion (SWE) induces a specific T cell IFN-γ response.	[85]
	IFNα	PRRSV KV	Significantly increases the levels of PRRSV-specific antibodies, neutralizing antibodies, IL-4, IFN-γ, and lymphocytes.	[86]
	IFN-γ and GM-CSF	PRRSV KV	Significantly increases neutralizing antibody titers, accelerates viral clearance, reduces clinical symptoms, and prevents highly pathogenic PRRSV infection.	[87]
	Toll-like receptor agonists	07V063	TLR9 agonist reduces viremia, and induces a non-antigen-specific IFN-γ and an anamnestic antibody response after a homologous challenge.	[88]
	Purified fraction of Albizia julibrissin saponins	CH-1R IAV	Elicits both Th1/Th2 and Tc1/Tc2 response.	[89]
	A PRRSV-specific IgM monoclonal antibody (Mab)-PR5nf1	VR2332	Significantly improves serum IFN-γ, overall survival rate, and cell-mediated immunity (CMI) after challenge with HP-PRRSV.	[90]
Nanoparticles (NPs)	VLP-NPs with heat-labile enterotoxin subunit B (LTB) and dimethyldioctadecylammonium bromide (DDA)	S1/17 MA2-2 0117 ORF5US	Induces high levels of IFN-γ-producing cells, IgG, IgA, and viral neutralizing titers; low levels of IL-10, PRRSV RNA, and macro- and microscopic lung lesions.	[93]
	Mannose modified gelatin nanoparticle (MnGNP)	TJ-F10	Induce maturation of MoDCs and significantly enhance the expression of markers on MoDCs, the secretion of cytokines in MoDCs, the activation and proliferation of T cells, and function to kill PRRSV-infected cells.	[94]
	PLGA-NPs	VR2332	Increases the number of NK cells and γδ cells, secretion of IFNα and cytokines; response of CD8^+^ T cells and IFN-γ, and protection against homologous and heterologous challenge.	[95,96]
	PLGA-NPs with M. tuberculosis WCL	VR2332	Elicit strong antibody and cell-mediated reactions; enhanced heterologous protection.	[97,98]
Modification of viral proteins	Deglycosylation of GP5	FL12	Enhances the immunogenicity of the inactivated PRRSV, and double administrations confer protection against the homologous challenge.	[99]

## 5. Vector Vaccines

Viral vectors are a vaccine platform that relies on recombinant viruses to deliver selected specific immunogens to the host. The viral vector-based vaccine has the advantages of being safe, easy to manufacture, low cost, and the capacity to carry larger gene segments. However, there are disadvantages as well, like unstable exogenous gene expression and virulence recovery. Using genetic engineering technology, a live vector vaccine is produced by incorporating the recombinant PRRSV antigen gene into a living vector that can generate exogenous viral protein [100]. The vaccine can trigger a particular immune response following direct immunization of animals. Modified vaccinia virus Ankara (MVA), Newcastle disease virus, herpesvirus, porcine pseudorabies viruses (PRVs), adenovirus, etc., are the common viral vectors utilized in the development of PRRSV vaccines.

The modified vaccinia virus Ankara is an attenuated vaccinia virus strain with multiple alternative advantages: a high safety profile, the easy insertion of exogenous genes, transient expression, and the induction of both humoral and cellular responses. GP5 and M proteins of PRRSV have been expressed in MVA with different patterns to construct recombinant vaccines. The vaccine rMVA-GP5/M can improve the humoral and cellular responses, whereas rMVA-GP5 and rMVA-M do not.

Newcastle disease virus (NDV), which belongs to the Paramyxoviridae family, has been extensively utilized as a vaccine vector for preventing diseases in both humans and animals through reverse genetics technology. NDV has been used to express the HP-PRRSV GP5/GP3-GP5 protein to form recombinant vaccines, which are proven to be safe for immunized pigs. Compared with the commercially available vaccine, the vaccine can induce higher titers of neutralizing antibodies and IFN-γ, showing potential as a candidate vaccine against the epidemic of PRRS.

Herpesvirus is also widely used as a vaccine carrier because of its ability to induce high levels of T cells against encoded heterologous antigens. Bovine herpesvirus-4 (BoHV-4) is an attenuated herpesvirus-based vector that is used to express M and NSP5 fusion protein. Prime-boost immunization of pigs induces strong IFN-γ responses and reduces tissue damage but has negligible effects on control of viral load [101]. Pseudorabies virus (PRV) is an alphaherpesvirus of pigs, and attenuated strains of PRV have been utilized to control Aujeszky’s disease. Zhao et al. construct a recombinant PRV, which co-expresses the GP5 and M proteins of NADC30-like PRRSV stably. It induces both humoral and cellular immune responses specific to PRV and NADC30-like PRRSV, appearing to be a promising candidate vaccine.

Baculovirus has been explored as a promising vector for PRRS vaccines because of the simplicity of expressing foreign antigens and ability to infect mammalian cells without inducing cytopathic effects. Recombinant baculovirus co-expressing GP5 and M protein of PRRSV are constructed using a pseudotyped baculovirus with the glycoprotein of vesicular stomatitis virus (VSV-G) on the envelope as vectors under the transcriptional control of two separate cytomegalovirus immediate early enhancer/promoters. IFN-γ and PRRSV-specific neutralizing antibodies are produced in a dose-dependent manner by the recombinant baculovirus [102].

Recombinant adenoviruses (rAd) have been used as a vector to express PRRSV proteins, such as GP3, GP5, GP3-GP5, and GP5-M in several studies, which all enhance the induction of neutralizing antibodies and cellular immune response against PRRSV [103,104,105,106]. As vaccine vectors, rAd have attractive properties of high titers, easy insertion, long-term storage at 4 °C, infection of multiple host, tissue, and cell types, and the induction of strong immune responses when administered orally or intranasally, potentially bypassing pre-existing immunity against the vector [107].

The creation of infectious clones of PRRSV has made it possible to modify viral genomes in certain ways and produce mutant viruses. Porcine circovirus type 2 (PCV2) capsid protein gene was the first foreign genes to be expressed via PRRSV from dedicated subgenomic RNAs, demonstrating the potential use of PRRSV as a vaccine vector for swine pathogens [108]. Subsequently, a recombinant PRRSV vector vaccine candidate, rPRRSV-E2, expressing classical swine fever virus (CSFV) E2 protein, is demonstrated to protect piglets against lethal challenge of HP-PRRSV and CSFV [109]. Further research has shown that effective efficient protection against the challenge of heterologous circulating NADC30-like strain can be offered by rPRRSV-E2 [110].

Although viral vectors have been utilized in research for PRRSV vaccines, they have drawbacks of high production costs, low efficiency, and the potential for the vector virus to trigger an immune response that may reduce the vaccine’s effectiveness. However, given the increasing diversity of PRRSV, vector vaccines that can be easily modified and to express transgenes may be more suitable. In addition, viral vector vaccines show promise for vaccination using generic or focused antigens. Unlike viral proteins, which mutate rapidly, the viral receptors are conserved molecules and, therefore, offer attractive targets for therapeutic and prophylactic intervention. The scavenger receptor cysteine-rich domains 5–9 (SRCR59) of CD163 and the first four Ig-like domains of sialoadhesin (Sn4D) can act as the soluble viral receptors (SVRs) of PRRSV. Pigs can be protected against deadly PRRSV infections by co-injection with SVR-expressing rAd vectors [111,112]. Furthermore, expressing multiple antigens using PRRSV-based vectors will be especially helpful for creating a new generation of multivalent vaccines against mixed infection in pigs. Finally, unlike subunit vaccines, vectored vaccines produce antigens for weeks following inoculation, helping sustain a durable memory immune response. Therefore, with advancements in biotechnology, viral vector vaccines are expected to address the limitations of current PRRSV vaccines.

## 6. Subunit Vaccines

Protein subunit vaccines are based on viral proteins or peptides that are systemically expressed utilizing a variety of expressing systems, such as bacteria, yeasts, insects, and mammalian cells [113,114,115]. These vaccines have several advantages, including effectiveness, safety, and affordability.

The baculovirus gene has been used successfully to manufacture vaccines as its powerful promoter that can generate large numbers of foreign proteins and can perform protein processing and modification to promote optimal protein folding. Shortly after the discovery, PRRSV proteins expressed by baculoviruses have been investigated as a potential subunit vaccine (Table 5). Plana et al. conduct an animal immunity test and discover that GP3 and GP5 can provide specific protective effects [116]. Then, researchers have developed a polycistronic baculovirus surface display system to simultaneously express multiple PRRSV proteins and increase their expression levels to improve the immunogenicity of the subunit vaccine. The ectodomain of His-tagged GP2, GP4, and GP5 are simultaneously expressed with the C-terminal domain of a baculovirus glycoprotein, gp64, and displayed on the cell membrane of Sf-9 for preparation of subunit vaccines against PRRSV. The subunit vaccine induces a high level of IL-4, IFN-γ, ELISA-specific antibodies, and neutralizing antibodies. It indicates that the polycistronic baculovirus surface display system is a useful tool for the preparation of subunit vaccines of PRRSV [117].

Plants are also an expression system that can be scaled up quickly and easily to produce large amounts of recombinant proteins and have more sophisticated protein processing machinery than many other protein production platforms, which are considered as a promising alternative to conventional platforms. Several studies have shown that transgenic plants oral subunit vaccines (Table 5), such as GP5 transgenic tobacco and banana leaves are effective in generating immune responses to PRRSV [118,119]. To overcome the poor efficacy and safety of current vaccines, Chul et al. generated transgenic Arabidopsis plants expressing codon-optimized and transmembrane-deleted recombinant glycoproteins. Pigs fed with the transgenic plant had high titers of PRRSV-specific antibodies, as well as reduced viremia and viral loads after being challenged with PRRSV [120]. Plant tissue can be lyophilized and fed to the animals mixed into livestock feed, which is technically easy and can avoid the requirement to purify the recombinant proteins. Vaccines administered orally can directly increase the secretion of IgA in mucosal tissues of the intestinal tract. Since PRRSV infects mucosal tissues, IgA antibodies are probably better suited for interrupting the initial infection of the pigs.

However, the limited antigen quantity of subunit vaccines leads to a lesser memory immunological ability, a shorter immunogenicity endurance, and a weaker ability to trigger broad immunity. These vaccines usually require repeated doses compared with MLV vaccines. Therefore, it is necessary to implement effective measures to enhance the immunogenicity of subunit vaccines (Table 5). Peng et al. have studied the immune-enhancing effect of natural adjuvants and find that the cs/bacillus adjuvant and Taishan Pinus massoniana pollen polysaccharide (TPPPS) adjuvant are candidate immune adjuvants to more efficiently prevent and control PRRS [121,122]. In addition, porcine CD40 ligand (CD40L) can effectively increase humoral and cell-mediated immune responses of GP3 and GP5 protein [123].

Nanoparticles shield antigens from degradation by protein hydrolysis, prolong the bioavailability, and maintain a slow and sustained release of antigens, helping induce a better immune response. PRRS-VLPs containing up to five viral surface proteins (GP5-GP4-GP3-GP2a-M) are generated and entrapped in PLGA nanoparticles and co-administer intranasally with a potent adjuvant M. tuberculosis WCL. The PRRS-VLPs boost IgG and IFN-γ production and reduce the lung viral load [124]. Unfortunately, viremia can be exacerbated by intranasal immunizations of pigs with PRRSV VLPs and VLPs plus the 2′, 3′-cGAMP VacciGrade adjuvant [125]. The first and most important step in successfully inducing mucosal immunity is the efficient delivery of antigens through oral vaccination. Membranous/microfold cells (M cells) in the mucosa can transport internalized antigens without degradation and thus are crucial for triggering antigen-specific mucosal immune responses by promoting secretory IgA production. UEA-1/PLGA NPs, prepared using modified PLGA nanoparticles with Ulex europaeus agglutinin 1 (UEA-1), are an oral vaccine delivery system and efficiently delivered by M-cells to induce the markedly increased release of IgA intestinal and IgG in serum [126]. Hui et al. have constructed a GP5m-ferritin nanoparticle vaccine with fusion expression of modified GP5 (GP5m) and ferritin (Ft) by using a baculovirus system. The GP5m-Ft elicits higher serum antibody titers and enhanced specific T-lymphocyte immune responses. Pigs vaccinated with GP5m-ferritin exhibit significantly lower mean rectal temperatures, respiratory severity, viremia, and lung lesions after the challenge compared to unvaccinated pigs [127]. Piglets are well protected by GP5m-Ft nanoparticles from an HP-PRRSV challenge [128]. These results also imply that nanovaccines are a promising vaccine candidate for controlling PRRS.

Multi-epitope vaccines are a unique design concept based on a number of overlapping antigenic epitopes. Immunoinformatics methods are usually used to predict the immunogenic T- and B-cell epitopes, which are attached using proper linkers to make an epitope-rich peptide in a multi-epitope vaccine design. It has unique advantages in cellular immunity, which can effectively cope with the variation of pathogenic microorganisms and many unfavorable factors in immune response. This strategy may be a promising approach against PRRSV. Two multi-epitope subunit vaccines were designed based on the conserved B cell epitopes of viral proteins, with the addition of the N-terminal of porcine heat shock protein gp96 (Gp96N) as the adjuvant [129]. A multiepitope peptide (PTE) is constructed based on 10 PRRSV-specific CTL epitopes, which are identified through ELISA and fused with a modified porcine Fc molecule to create the recombinant protein [130]. All the combinations induced high titers of neutralizing antibodies and IFN-γ [129,130]. Another vaccine is constructed using a reverse vaccinology technique based a Pseudomonas exotoxin. The chimeric subunits consist of PRRSV conserved epitopes (including ORF1b, ORF7, and the heterodimeric complex of GP5 and M protein), a ligand moiety, a Pseudomonas exotoxin A deleted domain III (PE (ΔIII)), and a carboxyl-terminal moiety that contains a polypeptide with amino acid sequence KDEL. Pigs received the vaccinations exhibit robust and rapid neutralizing antibodies and IFN-γ responses, and a field trial evaluating the immune response of the vaccine to pregnant sows showed that it not only improved the reproductive performance of pregnant sows but also activated maternal immune protection to prevent piglets from causing viremia [131]. Another study uses immunoinformatics to identify 12 B-cell epitopes, 6 CTL epitopes, and 5 HTL epitopes of GP3 and GP5 proteins, which are fusion-expressed with the addition of a 50S ribosomal protein L7/L1 molecular adjuvant. However, the protective effect of the vaccine candidate needs further research [132]. A subunit vaccine with specific immune responses for different PRRSV strains will be designed.

**Table 5:** The studies of PRRSV subunit vaccines.

Antigens	Expression System	Modify	Pigs Age	Adjuvants	Route	Results	References
GP3 and GP5	Baculovirus/Sf9 cells	/	pregnant sows	/	IM	Provide specific protective effects	[117]
GP2, GP4, and GP5	Baculovirus/Sf9 cells	Ectodomain of fusion proteins	3 weeks	Montanide ISA201	IM	Induces elevated levels of IFN-γ, IL-4, neutralizing and ELISA-specific antibodies.	[118]
GP5	plant binary vector pGKU/tobacco leaves	/	6 weeks	/	Orally	Triggers specific mucosal, humoral, and cellular immune responses	[118]
GP5	plant binary vector pGKU/banana	/	6 weeks	/	Orally	Causes significantly low levels of viraemia and tissue viral load.	[119]
GP4 and GP5	plant transformation vector pGreenII0229/Arabidopsis	Codon-optimized and transmembrane-deleted	4 weeks	/	Orally	Has high levels of PRRSV-specific antibodies, pro-inflammatory cytokines (TNF-α and IL-12), and IFN-γ-producing cells, and low levels of regulatory T cells.	[120]
GP5	Escherichia coli	combined with two hydrophilic fragments by a four-aa-linker (GGSG)	3 weeks	Astragalus/Bacillus	IM	Enhances humoral immune and cell-mediated immune responses; has low viremia, slight clinical signs, and less pathological lung lesions.	[121]
GP5	Escherichia coli BL21	combined with two hydrophilic fragments by a four-aa-linker (GGSG)	3 weeks	Taishan Pinus massoniana pollen polysaccharide (TPPPS)	IM	Has high titers of specific antibodies, neutralizing antibodies, T lymphocyte proliferation, and the percentage of the CD3(+) T lymphocyte subpopulation; has low viremia, few clinical signs, and few pathological lung lesions.	[122]
GP3 and GP5	adenoviruses/HEK-293A cells	/	3 weeks	porcine CD40 ligand	IM	Provides significant high specific anti-PRRSV ELISA antibody, neutralizing antibody, IFN-γ, and IL-4; shows lighter clinical signs and lower viremia following homologous challenge.	[123]
GP5, GP4, GP3, GP2a and M proteins	Baculovirus/Sf9 cells	Entrapped in PLGA nanoparticles	4~6 weeks	M. tuberculosis WCL	IN	Boosts IgG and IFN-γ production; a two-log reduction in the lung viral load; offers partial protection after challenge with heterologous virus.	[124]
N, M, GP5 and E proteins	Baculovirus/Sf9 cells	Entrapped in VLP nanoparticles	3 weeks	2′,3′-cGAMP VacciGrade™ (Invivogen, USA)	IN	Enhanced viremia associated with IFN-α, IFN-γ, and IL-10.	[125]
GP5	pcDNA3.1/Hela cells	Entrapped in PLGA nanoparticles of modified GP5 (inserting a Pan DR T-helper cell epitope (PADRE) between the neutralizing epitope and the decoy epitope)	3 weeks	Ulex europaeus agglutinin 1 (UEA-1)	Orally	Increased serum IgG levels and augmented intestinal IgA levels.	[126]
GP5	Baculovirus/Sf9 cells	Fusion expression of modified GP5 (Replace the decoy epitope of GP5 by the neutralizing epitope and replaced the asparagine triplet (AAC) at positions N34, N44, and N51 of GP5 with the alanine triplet (GCC)) and ferritin, and entrapped in VLP nanoparticles	2 weeks/4 weeks	ISA-201/aluminum hydroxide adjuvant	IM	High serum antibody levels, neutralizing antibody titers, T lymphocyte proliferation index, and IFN-γ levels; effectively protected piglets against a highly pathogenic PRRSV challenge.	[127,128]
GP4, GP5, and N proteins	synthesized	Fusion expression of two B-cell epitopes, seven T cell epitopes, with a Pan DR T-helper cell epitope	4 weeks	N-terminal 22-355 aa of heat shock proteinGp96 (Gp96N)	IM	Causes mild clinical symptoms, low viremia, and little pathological lesions in the lungs following challenge with HP-PRRSV, but cannot provide lasting and effective protection against HP-PRRSV infection.	[129]
ORF7, ORF1b, and GP5&M proteins	pET23 vector/Escherichia coli	Fusion expression of 10 PRRSV-specific CTL epitopes in ORF7, ORF1b, and ORF6&5, with a modified porcine Fc	4 weeks/sows	Pseudo monas exotoxin A (PE)	IM	Induces PRRSV-specific INF-γ cellular immunity and neutralizing antibodies in pigs; enhances sow reproductive performance and activates maternal immune defenses to prevent piglets from inflicting viremia.	[130]

## 7. Nucleic Acid Vaccines

Nucleic acid vaccines include DNA and RNA vaccines, which directly introduce the viral gene fragment (DNA or RNA) into the animal or human cells to generate the antigen protein through the protein synthesis system of the host cell and induce the host to produce the immune response to the antigen protein, thereby preventing and treating diseases. DNA vaccines use DNA vectors to insert a piece of specific gene segments encoding antigens, and one of their benefits over mRNA vaccines is better thermal stability and less refrigeration requirements. Nevertheless, concerns have been raised previous about the potential risk of integrating the host genome, although it is merely a theoretical possibility. The DNA vaccine is designed to minimize the possibility of genetic integration. In contrast to the linear chromosomes of host genome, the DNA utilized in vaccines is typically in the form of a circular plasmid, and the amount of DNA delivered by a DNA vaccine is usually very small, which lowers the chance of integration. According to the results of numerous preclinical and clinical investigations the risk of integration is low [133,134]. To guarantee the safety of DNA vaccines, it is crucial to carry out more monitoring and research. The mRNA vaccine encodes antigen genes as an mRNA or self-amplifying mRNA, which is translated into the cytoplasm of the host cells after inoculation. The vaccine utilizes in vitro transcription without the need to be multiplied in bacteria or cell culture and does not alter or affect the host genome, so the production of mRNA vaccines is easier and shorter, with less risk of insertion. However, to overcome the instability and increase the translational efficiency of the mRNA vaccine, coding sequence optimization, modified nucleotide screening, and delivery system optimization are required.

As a third-generation vaccine, nucleic acid vaccines have the characteristics of low cost, rapid production process and easy to scale, flexible and efficient research and development compared with the previous two generations of vaccines, and the ability to induce sustained humoral and cellular immune responses [135,136].

### 7.1. DNA Vaccines for PRRSV

Several studies have revealed that DNA vaccines encoding the GP5 induce humoral and cellular responses in piglets. For example, a plasmid that encodes GP5 is designed to be controlled by a human cytomegalovirus promoter, which can induce anti-GP5-specific neutralizing antibodies and prevent the occurrence of viremia and the development of typical macroscopic lung disease after challenge with the virulent IAF-Klop strain of PRRSV.

Although GP5 is the most important immunogenic protein, increasing evidence that GP5-based vaccines are not fully protective. Several measures have been taken to enhance the immunogenicity of these vaccines. DNA vaccines encoding GP5 and porcine glutathione peroxidase-1 (GPX1), cytotoxic-T-lymphocyte-associated protein 4 (CTLA4), and cytokines such as IL-2, IL-4, IL-12, IL-18, IFN-α/γ, and IFN-λ may improve the immune efficacy of DNA vaccines against PRRSV [137,138,139,140]. Besides, it is thought that the presence of a non-neutralizing decoy epitope and high glycosylation close to the neutralizing epitope is responsible for the incapacity to elicit strong protective immunity. Based on the native ORF5 gene of HP-PRRSV, Li et al. synthesized an ORF5 gene, which optimized its codon usage for expression in mammalian cells, inserted a Pan DR T-helper cell epitope between the neutralizing and non-neutralizing decoy epitope, and mutated four putative N-glycosylation sites. The DNA vaccine causes significantly enhanced expression of GP5-specific antibodies, neutralizing antibodies, IFN-γ level, as well as lymphocyte proliferation responses in mice and piglets, compared with DNA vaccine expressing the native GP5, which will be useful to facilitate the development of efficient vaccines against PRRSV in the future. In addition, mosaic DNA and vaccinia (VACV) vaccines are developed to improve protection against heterologous PRRSV strains. A GP5-Mosaic vaccine using DNA-prime/VACV enhancement induced cellular responses and increased levels of neutralizing antibodies against both VR2332 and MN184C PRRSV strains, indicating that GP5-Mosaic vaccines confer protection in pigs against heterologous viruses [141]. Co-expression of GP5 and GP3/M/N as fusion proteins with appropriate adjuvants can arouse better immunogenicity than expressing GP5 alone [140,142,143,144]. The heterodimer of GP5 and M protein is the leading target for the creation of a new generation of vaccines to prevent PRRSV infection [142]. Following the PRRSV challenge, a gradual increased level of neutralizing antibodies titers and a decreased level and short period of viremia are detected in the pigs inoculation with the DNA construct fusion expression of GP5 and M proteins via a Glycine-proline-glycine-proline linker, which can minimize the conformational changes in tertiary structure and provide flexibility of the peptide chain [145]. However, an optimal delivery method remains to be determined. Scientists have studied alternative ways and methods of using hypodermic needles to deliver DNA vaccines instead of the usual subcutaneous and intramuscular routes. Percutaneous, mucosal, microneedle applications, jet injection, and electroporation, have all been investigated to improve the immunogenicity of DNA vaccines [146,147]. Furthermore, using porcine endogenous retrovirus (PERV)-modified baculovirus as a delivery vector to deliver GP5 and M DNA induces comparatively high humoral and cellular immune responses.

### 7.2. RNA Vaccines for PRRSV

Recently, a range of messenger RNA (mRNA) vaccines have been constructed to combat COVID-19, marking a significant advancement in the development of mRNA vaccines, which has significant potential for the prevention and management of the PRRS epidemic. In 2023, Thomas et al. successfully engineered a self-replicating replicon RNA (RepRNA) construct encoding PRRSV N protein using a Coatsome-replicon vehicle based on Coatsome^®^ SS technologies. The RepRNA constructs are highly immunogenic and provide a paradigm for antigenic expression with potential application to combat viral diseases [148]. Cui et al. have constructed an mRNA vaccine against an HP-PRRSV strain, which significantly stimulates cellular and humoral immune responses in mice. Notably, when administered in high doses, the GP5-mRNA exhibits an immune response similar to that of a currently available commercial vaccination [149]. The study offers a strong basis for the investigation of PRRS mRNA vaccines despite the fact that it only involves mouse animal experiments and does not include pig immunization and challenge protection tests.

## 8. Discussion and Some Strategies

Strategies such as management, biosecurity, and vaccination have been used to control PRRS outbreaks, with varying degrees of success. The pattern of persistent, subclinical infections with sporadic epidemic outbreaks, the large viral heterogeneity, and weak antibody responses make controlling PRRS challenging [150]. Vaccination is currently the most economical and dependable method available, even though new vaccine technologies and improvements to the current vaccinations are required.

Vaccine preparation

Many studies have been conducted to explore new approaches to PRRS vaccine preparation, as mentioned above. For example, a new generation of adjuvants is being developed, including nanoparticle-based adjuvants, liposomal adjuvants, immune-stimulating adjuvants, bacterial toxin, etc. In particular, toxin molecules extracted from bacteria such as Escherichia coli (for heat unstable toxin) or Vibrio cholerae (for cholera toxin), play an important role in infectious diseases to hijack mammalian cells and manipulate host immune responses. The ability to deliver toxin adjuvants by both systemic and mucosal routes combined with its capacity for simple admixing with many antigen types (live, killed, protein, DNA, etc.) allow for tremendous flexibility in vaccine design. Bacterial toxins have been used as adjuvants to construct PRRS vaccines, and have been demonstrated to boost immune responses and reduce viremia [93,124,151]. By combining different types of adjuvants, a synergistic effect may be achieved, resulting in a stronger and more durable immune response against PRRSV.

In addition, genetic engineering techniques, such as reverse genetics, could lead to the development of novel multivalent or broad-spectrum vaccines. Reverse genetic systems, which have been described in several publications, may be useful tools because they can easily induce modifications and changes to the viral genome. The strategies include modification of viral proteins, establishment of chimeric viruses, codon anti-optimization, etc. For example, chimeric viruses constructed with different parental viruses (VR2385, VR2430, MN184B, JXA1, FL-12, and NADC20) have broadened cross-neutralizing antibody activities against heterologous PRRSV strain. De-optimize codon pairs in the GP5, nsp1, or nsp9 gene and the recombination virus could protect pigs against homologous and heterologous PRRSV assaults. They may help in the design and development of recombination-deficient, genetically stable PRRS MLV strains [11]. A combination of these strategies may help create an effective and safer live vaccine. For instance, removing immunosuppressive domains from the viral genome, and the codon optimization of the sites prone to mutation or recombination, as well as a suitable adjuvant, appear to construct a vaccine that can offer broader protection against hetero-viral strains and avoid the risk of viral recombination.

Finally, the considerable genetic diversity and the lack of cross-protectivity is one of the most evident obstacles to vaccine development. Referring to bacterial vaccines, new vaccine developments have narrowed the spectrum to individual, epidemiologically relevant strains known to possess resistance to a particular type of antibiotic or toxin, rather than focusing on broad coverage of strains. Thus, investigating strain-specific vaccines may be desirable, and eschewing the idea of vaccines covering a wide range of strains may facilitate targeted vaccine development, such as developing vaccines that target specific epitopes.

Vaccine combination immunization

Without major technological advancements, vaccines based on novel strategies are not yet ready for practical application and are unlikely to replace MLVs without major technological breakthroughs. Therefore, MLV vaccines should be used more carefully for PRRSV control, especially to avoid the use of two or more PRRS MLV vaccine strains on a farm. Moreover, the optimal immunization schedule should be prepared to strictly control the vaccination dose and time, and select an appropriate vaccination route. In addition, although other vaccines may not be sufficient to provide complete protection, they can be used as a supplement to MLVs to enhance heterologous protection because of their safety. Administering a DNA vaccine encoding a truncated PRRSV N protein two weeks before MLV immunization can increase neutralizing antibody titers and PRRSV-specific IFN-γ levels [152,153]. Although the combination of DNA vaccines and MLVs does not achieve total protection, it is highlighted that using a heterologous prime-boost vaccination regimen-where DNA/RNA/protein can be incorporated with other vaccine candidates-has the potential to boost anti-PRRSV immunity. Inactivated vaccines can also be used as a booster to protect synergistically with MLV vaccines. The vaccination of sows with the MLV vaccine during the 8 weeks of gestation, followed by another commercial inactivated vaccine 3 weeks before farrowing, is effective at reducing the incidence of PRRSV. In addition, piglets are partially protected by the combination of the MLV vaccine and inactivated vaccine with increased weight gain, and reduced pathological lung loss and viremia [154]. This approach may ultimately lead to the induction of complete protection against PRRSV reducing the use frequency of MLV vaccine to reduce the possible occurrence of safety events such as recombination.

Vaccine administration

Vaccine administration is one of the most critical issues in animal vaccination. A range of traditional and alternative administration routes are used for vaccination of pigs. Rapid immunization, minimal animal suffering, cheap labor costs, and non-invasiveness make it the ideal method of administering vaccines. The common methods of vaccine administration include intramuscular, intradermal, and subcutaneous injections. Different inoculation routes induce different intensities of immune response. Muscle tissue contains relatively few resident immune cells. However, after intramuscular vaccination, immune cells are significantly recruited, and local inflammatory reactions are induced. In addition, most adjuvants are well absorbed in muscle tissue, such as lipid-containing adjuvants. The skin is rich in a large number of immune-related cells that are involved in the initial stages of inflammation, repair, and immune response. Dermal vaccination can induce similar antibody titers using doses lower than those used for the intramuscular route. The intradermal injection may reduce the levels of IL-10 and increase IFN-γ-SC levels, thereby increasing the efficacy of the vaccine [155]. In addition, needle-free injection or jet injection is a new injection method, which diffuses the drug liquid into the skin by relying on high-speed airflow, that is, the drug injection does not use needles, and there is no needle handling problem of traditional syringes, and can avoid the cross-infection that may be caused by traditional syringes. Continuous explorations in the direction of needle-free administration, and intradermal vaccination combined with needle-free syringes through high-pressure jet or diffusion injection, may reduce the shedding of vaccine viruses and reduce the iatrogenic transfer of pathogens among animals.

## 9. Conclusions

PRRS has been a severe epizootic disease with great economic significance for more than 30 years, affecting the global swine industry. Unfortunately, despite continuous research aimed at understanding the pathogenesis and vaccinology of PRRSV, a successful vaccine for preventing PRRSV remains elusive. MLVs produced from a particular strain of PRRSV provide considerable protection against homologous viruses, but only partial protection against heterologous strains. Outbreaks still occur, even in herds that receive regularly vaccinations, because of the high genetic variety and unpredictability of PRRSV. These underscore the two major concerns of heterologous cross-protection efficacy and safety in PRRS MLV vaccines, and inactivated vaccines. Although other vaccine approaches, including virus-vectored vaccines, subunit vaccines, or nucleic acid vaccines have been explored, it is still unclear if these would be useful as alternatives to the currently used PRRS commercial vaccines. Modern advanced biotechnology can be used to explore ways for eradicating PRRS, such as modifying MLV vaccines based on reverse gene manipulation techniques, using new adjuvants and antigen delivery systems to enhance vaccine-induced immune responses, or changing vaccination routes. Furthermore, further elucidation of the mechanisms of PRRSV mutation, recombination, and immune response is needed, which will contribute to the development of an effective, safe, and stable vaccine.

## Figures and Tables

**Table 1:** Key challenges for PRRS vaccines and some possible solutions.

Characteristics of a “Perfect” PRRS Vaccine	Key Challenges for PRRS Vaccines	Possible Solutions
Broad-spectrum protection	High genetic variability	Reverse vaccinology
The vaccine should provide protection against all known genetic variants and subtypes of the PRRSV.	PRRSV is highly diverse, meaning that the main targets for the host immune response have significant differences between strains. The virus constantly mutates, leading to the emergence of new variants that escape the immunity conferred by existing vaccines. And the new PRRSV variants can quickly spread and cause disease outbreaks, even in herds that have been previously vaccinated.	Modification of viral proteins: mutating or modifying the genes involved in immune modulations to enhance immune responses. Chimeras: constructing chimeric vaccines of several strains can broaden the antigenic cross-reactivity. Codon pair de-optimization: the marked sequence divergence between codon pair de-optimized viruses and circulating strains reduces the chances of recombination and virulence reversal.
Highly effective immunological protection.	Insufficient immune protection.	Adjuvant- and nanoparticle-based vaccines.
The vaccine should quickly and strongly stimulate both the innate and adaptive immune responses, and also induce a long lasting memory response.	Immune evasion: inhibit interferon (IFN) production and signaling; infect and modify the function of antigen-presenting cells such as macrophages and dendritic cells. Dysregulation of cellular immune responses: causes thymus lesions and a delayed induction of effector T cells. Delayed production of neutralizing antibodies (NAs): can be detected at around 28–42 days after infection when the viremia has subsided, but they cannot prevent the establishment of chronic infection, which lingers in tissues for months. Non-neutralizing antibodies and antibody dependence enhancement (ADE) [11,12,13].	Vaccine adjuvants: improve immunological effective and protective capabilities, and reduce the amount of immune substances to lower the cost of vaccine manufacturing. Nanoparticles: can protect the antigens from degradation and target them to specific immune cells; can also be modified to carry multiple antigens from different PRRSV strains; can be conjugated with adjuvants to enhance the immune response, providing a more effective vaccine solution for the highly variable PRRSV.
Safety	Safety concerns with PRRS MLV vaccines	Combined immunization or multivalent vaccine
The vaccine should have no risk of causing disease or any other adverse side effects; have no chance to revert to a virulent form for PRRS MLV vaccines.	Vaccine viruses shed and persist in vaccinated animals, which may lead to the spread of vaccine viruses to unvaccinated animals; vaccine viruses can cross the placental barrier in pregnant sows, thereby infecting the developing fetuses and subsequently transmitting the virus to naive newborn piglets during lactation; the vaccine virus may recombine with field strains or other vaccine strains, resulting in the creation of potentially novel genetically distinct PRRSV variants, which may increase virulence and disease incidence [15,16,17,18,19].	Combination of PRRS MLV vaccines and inactivated/subunit/DNA vaccines: MLV vaccines are required for effective primary immunization, and other vaccines are used for booster to maintain a prolonged immune response. Selection of antigens or epitopes: identify conserved antigens or epitopes across different PRRSV strains and designed vaccines to target the conserved regions, which is more likely to be effective against a wide range of PRRSV strains.
Ease of administration, good stability, and cost-effectiveness	Inadequacies in inoculation, storage, and cost	Vaccination in combination with other control strategies
The vaccine should be easy to administer, preferably through a single-dose injection or an even more convenient route such as intranasal or oral administration, and have a high level of stability during storage and transportation; the production costs should be low enough to ensure that it is affordable for pig farmers.	The efficacy of PRRS vaccines can vary depending on factors such as the pig’s age, immune status, and the prevalent PRRSV strain, leading to the need for additional booster vaccinations; the vaccines should be stored in a constant temperature environment between 2 and 8 °C to ensure the biological activity of the antigen; incorrect injection could lead to poor vaccine absorption, abscess formation, or even injection site reactions that may affect the heathy of pigs and the efficacy of vaccines; the development of effective PRRS vaccines requires extensive research and it is needed to constantly update the vaccine to match the evolving PRRSV strains, which further adds to the research and development expenses [20].	Appropriate vaccination strategies: MLV vaccines should be used more carefully for PRRSV control, especially to avoid the use of two or more live multiplex PRRSV vaccine strains in the location. Moreover, the optimal immunization schedule should be prepared to strictly control the immunization dose and the interval between booster immunization and select an appropriate vaccination route. Erea elimination: utilizing a combination of load, close, homogenise (LCH) with PRRSV-2 MLV, optimized pig flow, and “10 Golden Rules” for biosecurity management to successfully eradicate PRRSV from all 12 herds on the Horne Peninsula, Denmark [21]. And using an inactivated vaccine and performing segregated rearing of the offspring are successful in eliminating PRRSV in a large-scale pig farm during the National PRRS Eradication Programme of Hungary [22].

## Data Availability

Not applicable.
